# Maternal effects, flight versus fecundity trade-offs, and offspring immune defence in the Speckled Wood butterfly, *Pararge aegeria*

**DOI:** 10.1186/1471-2148-10-345

**Published:** 2010-11-10

**Authors:** Melanie Gibbs, Casper J Breuker, Helen Hesketh, Rosemary S Hails, Hans Van Dyck

**Affiliations:** 1 Behavioural Ecology & Conservation Group, Biodiversity Research Centre, Earth and Life Institute, Université catholique de Louvain (UCL), Louvain-la-Neuve, Belgium; 2 NERC Centre for Ecology and Hydrology, Maclean Building, Benson Lane, Crowmarsh Gifford, Wallingford, OX10 8BB, UK; 3 Evolutionary Developmental Biology Research Group, School of Life Sciences, Oxford Brookes University, Gipsy Lane, Headington, Oxford, OX3 0BP, UK

## Abstract

**Background:**

Maternal condition can generate resource-related maternal effects through differential egg provisioning, and can greatly affect offspring performance. In the present study, the speckled wood butterfly *Pararge aegeria *(L.) was used to investigate whether (after controlling for egg size) maternal age, and increased flight during the oviposition period, resulted in changes in egg provisioning and whether this contributed to variation in offspring performance, i) early in development (egg stage and early post-hatching development), and ii) later in larval development after being exposed to the model viral pathogen system; the baculovirus *Autographa californica *multinucleocapsid nucleopolyhedrovirus (AcMNPV).

**Results:**

Age-related changes in maternal egg provisioning were observed to influence egg stage development only. Flight-induced changes in maternal egg provisioning had direct consequences for offspring growth and survival across each life stage from egg to adulthood; offspring from forced flight mothers had lower larval masses and longer development times. Offspring with lower larval masses also had reduced survival after exposure to the viral pathogen.

**Conclusion:**

The present study demonstrates that a change in maternal provisioning as a result of increased flight during the oviposition period has the potential to exert non-genetic cross-generational fitness effects in *P. aegeria*. This could have important consequences for population dynamics, particularly in fragmented anthropogenic landscapes.

## Background

Maternal condition and/or the environment can generate resource-related maternal effects through differential egg provisioning, and can greatly affect offspring performance (for reviews see [1-4]). In insect species, a decline in resources available for reproduction often results in a decrease in egg size with maternal age [1]. This decrease in egg size has been shown to influence offspring performance, with small eggs having a lower hatching success [5-7], and producing offspring which have; a lower survival during the larval stage [8], slower development [9-12], smaller pupae and/or adults [9,10,13] and lower starvation tolerance [[14-16], but see [17]], or desiccation tolerance [18].

The oogenesis-flight syndrome hypothesis [19] predicts that, in flying insect species, physiological constraints caused by an overlap in resources used during flight and during oogenesis results in a resource allocation trade-off, with fewer resources available for egg production [[20,21], but see [22]]. Increased flight activity during the oviposition period has been shown to have an impact on resource allocation to egg size, with females that were forced to fly laying smaller eggs [7,23-25]. This suggests the potential for increased flight during oviposition to generate resource-related maternal effects and affect offspring performance.

Resource-related maternal effects may be difficult to measure via egg size alone [12]. Recent studies have shown that variation in offspring performance with maternal age may not necessarily be due to egg size *per se*, but may be due to changes in the relative proportion of different nutrients allocated to eggs, with egg composition varying independent of egg size [6,12,26,27]. Although increased flight during the oviposition period reduces egg size, currently it is not known whether flight also results in changes in egg composition [[7], but see [25]]. In general, maternal effects usually have their greatest impact during early development [4,28,29]. As such, changes in maternal egg provisioning due to, for example, maternal age or increased flight during the oviposition period, may affect embryonic development independently from their effects on egg size *per se*, influencing early offspring developmental traits such as; embryonic development time, and early post-hatching survival and development rates (reviewed in [30]).

Using the speckled wood butterfly, *Pararge aegeria *(L.), we have previously shown that mothers alter the size of eggs both, as they age and, in response to increased flight prior to the onset of oviposition [7]. Also, we have demonstrated that larvae hatching from small eggs have lower post-hatching resource availability and need to grow for longer on poor quality host plants to obtain the critical mass required for pupation [7]. In line with predictions from life history theory, there is good evidence (in both vertebrates and invertebrates) to suggest that deployment of the immune system is energetically costly and can result in trade-offs with fitness-related traits such as; development time and pupal weight (for a general review [31]; and for baculoviruses specifically; [32-36]). It is also known that effective resistance to pathogens or parasites is often dependent on resource availability (particularly carbohydrate and protein; [37]), because resource availability affects body condition and also immune system development [38]. Given that *P. aegeria *larvae hatching from small eggs have lower post-hatching resource availability [7] one may predict that these larvae also have a reduced capacity for effective immune system deployment. As such, we hypothesised that a decline in maternal provisioning of eggs with age and time spent flying, reduces the developing offspring's capacity for effective immune function.

Our aim in this study is to investigate whether, after controlling for egg size, maternal age *per se *and increased flight during the oviposition period influences offspring performance; i) early in development (egg stage and early post-hatching development), and ii) later in larval development after being exposed to the pathogen system, the baculovirus *Autographa californica *multinucleocapsid nucleopolyhedrovirus (AcMNPV). AcMNPV is a popular model system to study the effects of a viral infection, and associated immune response, on life history traits in Lepidoptera [35,36]. In the present study, costs associated with the immune response were estimated in terms of *P. aegeria *larval survival, development time and pupal mass.

## Methods

### Study species

Speckled wood butterflies, *P. aegeria*, are temperate zone butterflies that lay eggs singly on grass plant species from the family Poaceae [39]. Females mate soon after emergence and usually mate only once [40]. At eclosion they have no [41] or only a few (8-24; [42]) mature oocytes, and if mated on the day of emergence they usually start ovipositing 48 hr later on the third day of their life [7,25]. In female *P. aegeria *resources for reproduction are, to a significant degree, obtained during the larval stage and there is little opportunity to obtain more nitrogenous resources for reproduction through adult feeding [43] or nuptial gifts [44]. During nectar and honeydew feeding, adults imbibe a dilute aqueous solution of mainly sugar with minute quantities of amino acids and lipids [45,46]. Therefore, through adult feeding *P. aegeria *may gain extra carbohydrate (but not lipid and protein) resources to fuel somatic maintenance and flight (cf. [46]).

### Baculovirus production

A stock of *Ac*MNPV was obtained by dosing 3^rd ^instar *Trichoplusia ni *(Hübner) larvae. Viral inoculum was added to small plugs of artificial diet and larvae were maintained individually and allowed to feed overnight. Once the entire plug of diet was consumed, larvae were incubated in individual pots containing artificial diet until death due to virus. Viral cadavers were collected, macerated and the resulting suspensions filtered through sterile muslin to remove large particulate matter. Viral material was then purified using density gradient centrifugation [47]. The concentration of occlusion bodies in the viral stock was estimated by counting in an improved Neubauer haemocytometer and stored at -20°C until required. Dilution of the stock suspension was done in sterile distilled water to achieve a final concentration of 1 × 10^6 ^OB's μl^-1 ^for use in experiments.

### Butterfly breeding design

Eggs were collected from a large outbred laboratory population of *P. aegeria *(kept at 300-400 individuals per generation). This population originated from a woodland population in the south of Belgium (St. Hubert; established from 50 eggs) and, by the time of the experiment, had been reared in the laboratory for 12 generations. Newly hatched larvae (siblings, 4 per plant) were placed on potted host plants of *Poa trivialis *(L.) with access to *ad libitum *food and were reared until eclosion in a climate room under a regime (26°C, LD 16:8) that promotes direct development (i.e. no diapause). On the day of eclosion (i.e. day -1, between 9 and 12 h) 36 females from this laboratory stock were weighed (Ohaus Explorer balance; accuracy: ± 0.1 mg) and placed individually in netted cages (0.5 m^3^) along with a potted *P. trivialis *plant for oviposition and an artificial flower containing a 10% honey solution [48]. Later the same day (between 13.00 and 16.00 h) a non-sibling virgin male was introduced to the cage and the mating pair was left undisturbed for 24 h.

### Maternal treatment (forced flight versus no flight)

On the day after mating (i.e. day 0), females were assigned to one of two treatment groups: control or forced flight. Control females were left undisturbed in their cages until the first egg was laid (i.e. day 1 of oviposition). When the first egg was laid, the male was removed from the cage and the female was left to continue laying for a further 9 days (i.e. 10 days in total). On day 0, experimental females were removed from cages and forced to fly for 5 min at 26°C. These experimental females were placed individually into an empty netted cage (0.5 m^3^) and stimulated to fly by gently touching their legs with a fine-bristled paintbrush each time they alighted (after [7,25]). After forced flight, the experimental females were returned to their mating cages until the first egg was laid (i.e. day 1 of oviposition). When the first egg was laid, the male was removed from the cage and the female was left undisturbed in the cage to continue egg laying. On days 4 and 8 of oviposition the flight treatment was repeated so that in total each experimental female was forced to fly 3 times. Each day, the host plants were watered and fresh honey solution was provided. In total, 18 control females and 18 experimental females were set up. All 36 females mated successfully, started egg laying 24 h after mating, and laid viable eggs.

Each day of oviposition, eggs were collected from every cage and counted. These egg counts provided a measure of daily fecundity for each female. A random sample of eggs laid by each female on days 2, 4, 6, 8 and 10 of oviposition were selected, and placed individually into labelled Petri dishes (n = 283 in total; average of 7.7 ± 0.54 eggs per female). Each egg was photographed with a digital camera (Canon A720 IS; Canon, Japan). The resulting images were then analysed using Image J (available at: http://rsb.info.nih.gov/ij/) and the size of each egg was measured as a cross-sectional projection (mm^2^). This method provides a highly reliable measure of egg size in *P. aegeria *because there is a strong correlation between egg area and egg mass in this species [49]. These eggs were left undisturbed in Petri dishes until hatching (at 26°C, LD 16:8). Hatching success was recorded for each egg.

### Offspring life history: pre-viral inoculation

For each egg, the number of days between being laid and hatching was recorded and used as a measure of embryonic development time. Newly-hatched larvae were placed individually on enclosed potted host plants of *P. trivialis *and reared under a direct development regime (LD 16:8 h photoperiod at 18°C). Larval development and survival was recorded for each individual up to 21 days. At 21 days of larval development (when the larvae were in their second instar), all larvae were removed from their host plants, weighed (Ohaus Explorer balance; accuracy: ± 0.1 mg) and placed individually into labelled Petri dishes lined with filter paper moistened with sterile water. Larvae were then left for 24 h without food to ensure consumption of inoculated *P. trivialis *leaves (see below).

### Offspring life history: post-viral inoculation

After 24 h without food, half of the larvae from each female were assigned to one of two treatment groups: control or infected. Each larva in the control treatment group was given 5 × 1 cm pieces of *P. trivialis *leaves inoculated with 1 microlitre of sterile water. Each larva in the infected treatment group was given 5 × 1 cm pieces of *P. trivialis *leaves inoculated with 1 microlitre of 1 × 10^6 ^OB's ml^-1 ^suspension of AcMNPV [after [50]]. Previous bioassays with the model system AcMNPV verified that when inoculated with 1 × 10^6 ^OBml^-1 ^at 21 days of larval development, 67% of *P. aegeria *survived to eclose as an adult (n = 37/55, unpublished data). As such, this concentration of AcMNPV was used to ensure that we could examine the sub-lethal effects of viral infection for the majority of our experimental larvae. All larvae were left for 24 h to consume the *P. trivialis *leaves. To ensure accurate dosing [35], we starved the larvae before inoculation, and ensured that each larva consumed all 5 of their inoculated *P. trivialis *leaves within the 24 h period. Thus we ensured that each larva received a comparable dose of virus. After this inoculation period, each larva was returned to its own enclosed potted host plant of *P. trivialis*. Each individual was monitored daily up to pupation. Larval development time was recorded as the number of days from hatching from the egg to pupation. On the day after pupation (once the pupal case had hardened) each pupa was weighed (Ohaus Explorer balance; accuracy: ± 0.1 mg) and placed into an individual container until eclosion as an adult. Survival to pupation and survival to eclosion as an adult was recorded for each individual.

### Statistical analyses

Linear and generalised linear mixed effect (lme or glme) models were fitted where appropriate, by means of restricted maximum likelihood (REML), which produces unbiased estimates of variance and covariance parameters, with female (nested within flight treatment) being declared as a random factor. Likelihood ratio tests were conducted to compare different models (that contained the same set of fixed factors but different random or interaction effects) with each other. The final model only included significant interactions. Residuals were examined for non-linearity in all cases and for non-normality where appropriate. Analyses were performed both in R 2.10.1 (packages nlme and lme4; http://lib.stat.cmu.edu/R/CRAN/) and Statistica 9.1 with R functionality (Statsoft Ltd.). Significances for REML constructed models in R are estimated by means of t_df_-values (lme) and z-values (glme). The sign of either the t- or z-values is indicative of the relationship between the effect and the dependent variable (i.e. positive or negative).

#### Reproductive output

Reproductive output of the 36 females was assessed by means of daily fecundity (egg number for days 1 to 10 of oviposition), and egg size (for a random subsample of eggs from days 2, 4, 6, 8, and 10 of oviposition). These data were analysed by means of a lme model for repeated measures data. Fixed factors were day of oviposition and flight treatment. Female body mass was used as a covariate.

#### Offspring development

lme models were constructed to investigate how each of the four offspring traits, embryonic development time (in days), larval mass at 21 days of development (mg), total larval development time (in days) and pupal mass (mg) changed over the egg-laying period (i.e. as a function of maternal age), and whether there were differences in these traits between females that had been forced to fly and the controls (i.e. flight treatment was a fixed effect), with viral challenge as an additional treatment factor. In these models, day of oviposition (fixed effect) was thus an indicator of female age. Egg size (mm^2^), which is known to affect offspring development [7], was used as a covariate, as any relationship between offspring traits and maternal age may simply be due to the fact that females lay differently sized eggs as they age. Larvae that developed to the pupal stage could be sexed, and thus offspring sex was also added as fixed effect to the models for total larval development time and pupal mass.

#### Survival analyses

Survival (0 = dead, 1 = alive) from; i) egg stage to 21 days of larval development, ii) from 21 days of larval development (when a viral treatment was administered) till pupation, and iii) pupa to adult stage were all analysed using lme models with a logit link function (i.e. logistic regressions with random effects). As the overdispersion parameter, by which p-values and confidence intervals should be adjusted for overdispersion, was never significantly different from 1, overdispersion correction did not alter the model output. For survival from egg stage to 21 days of larval development fixed effects were day of oviposition and flight treatment. Egg size was used as a covariate. For survival from 21 days of larval development till pupation, the fixed effects were viral treatment, day of oviposition and flight treatment. Larval mass at 21 days was used as a covariate. Offspring sex was not included in these models as offspring that did not survive could not be sexed.

## Results

### Effects of maternal weight, age and flight treatment on egg size and egg number

#### Egg number

**Figure 1:**
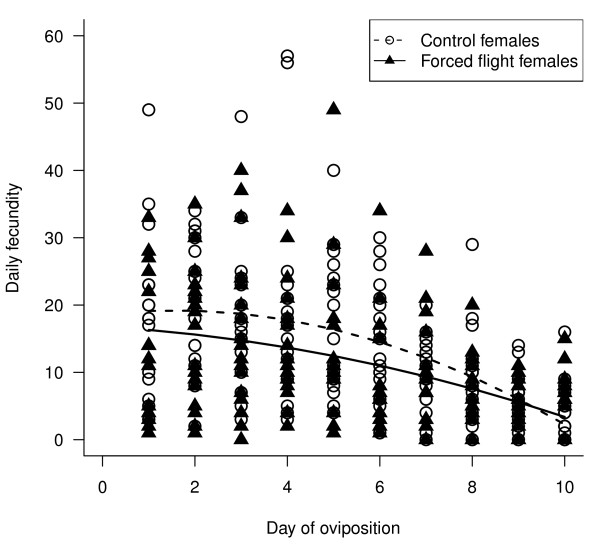
**The effect of flight on fecundity**. Number of the eggs laid (i.e. fecundity) by control females and females that had been forced to fly for each of the days of oviposition. Full lme model fitted was √*(egg number) = female body mass + flight treatment + day of oviposition + day of oviposition*^*2*^.

Egg number was affected by flight treatment and day of oviposition, and required a square root transformation for normality. Forced flight females laid significantly fewer eggs than control females over the whole of the 10-day oviposition period (control (± SE) = 140.55 ± 7.42, forced flight (± SE) = 117.11 ± 7.63; ANCOVA F_1,32 _= 4.80, p = 0.036); most notably in the first 6 days of oviposition (lme model t_33 _= -2.63, p = 0.013; Figure 1). Female body mass did not significantly explain variation in the number of eggs laid (ANCOVA F_1,32 _= 0.53, p = 0.47), nor did forced flight and control females differ in body mass (ANOVA F_1,33 _= 0.007, p = 0.93). The pattern of the number of eggs laid over time was not linear. Females generally reached a peak in egg production by day 3 after which the number of eggs laid declined (Figure 1, significant quadratic term; (day of oviposition)^2 ^t_322 _= -4.63, p < 0.001). There were no other significant interaction effects.

#### Egg size

**Figure 2:**
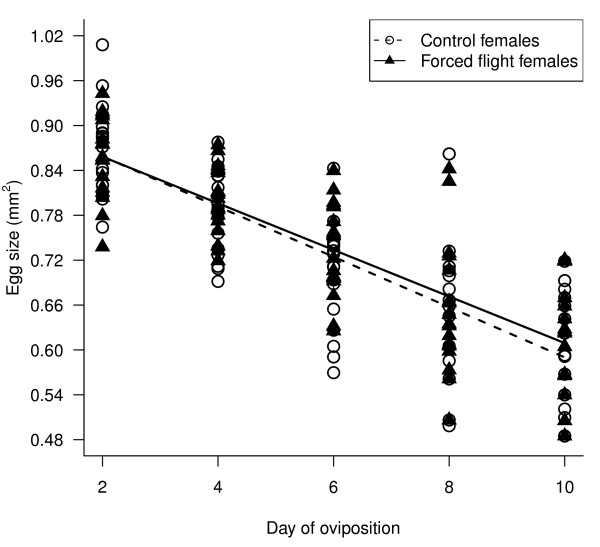
**The effect of flight on egg size**. Size (mm^2^) of the eggs laid by control females and females that had been forced to fly on days 2, 4, 6 and 8 of oviposition Full random intercept lme model fitted was *egg size = female body mass + flight treatment + day of oviposition*

As females aged, increasingly smaller eggs were laid; eggs laid later in the oviposition period are significantly smaller than those laid early. This decline was linear (day of oviposition t_143 _= -20.63, p < 0.001; Figure 2; non-significant quadratic term not included in final model). There were no differences in egg size between females forced to fly and the controls (flight treatment t_33 _= 0.62, p = 0.54; Figure 2). There was no effect of female body mass on egg size (female body mass t_33 _= 0.65, p = 0.52). There were no significant interaction effects.

### Maternal effects during early development (pre-viral inoculation)

#### Embryonic development time

**Figure 3:**
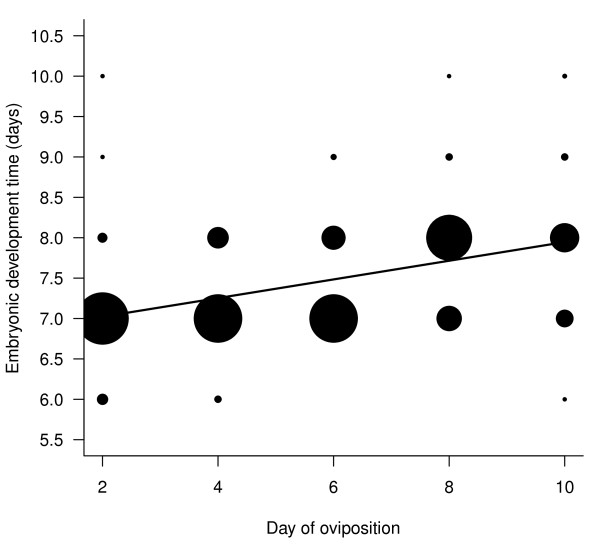
**Relationship between embryonic development time (in days) and day on which the egg was laid**. Size of the points is proportional to the frequency of the data points. Smallest point is 1 egg (e.g. egg laid on day 2 and taking 10 days to hatch) and largest corresponds with 40 eggs (e.g. 40 eggs laid on day 2 took 7 days to hatch). Full random slope and intercept lme model fitted was: *embryonic development time = egg size + day of oviposition + flight treatment*.

**Figure 4:**
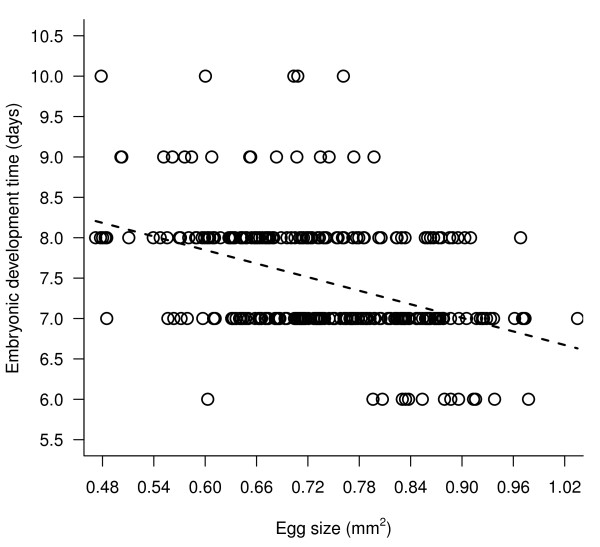
**Relationship between embryonic development time (days) and egg size (mm^2^)**. Full random slope and intercept lme model fitted was: *embryonic development time = egg size + day of oviposition + flight treatment*.

Both maternal age (i.e. day of oviposition) and egg size significantly explained variation in embryonic development (an increase with maternal age t_239 _= 2.55, p = 0.011, Figure 3; a decrease with egg size t_239 _= -2.82, p = 0.0052, Figure 4). Both effects were required in the model as the effect of maternal age on embryonic development time was not only as a result of egg size decreasing over the oviposition period (see earlier). Maternal flight treatment did not affect embryonic development time (t_239 _= 1.28, p = 0.28). There were no significant interactions.

#### Larval mass at 21 days of development

**Figure 5:**
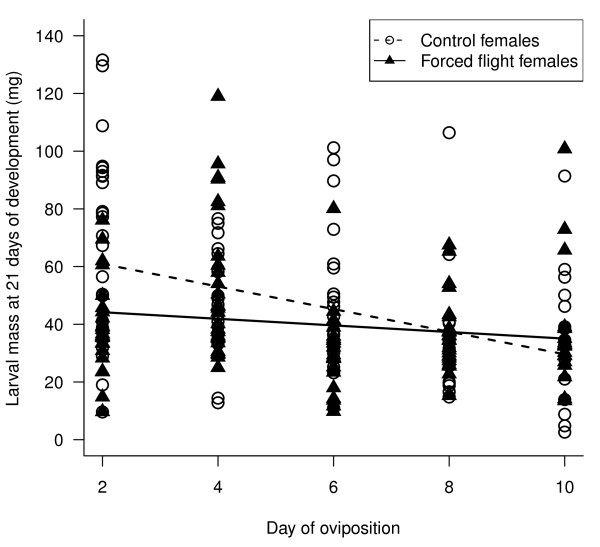
**Relationship between larval mass at 21 days of development (mg) and the day on which the egg was laid**. Larvae had mothers that either had been forced to fly or were controls as indicated. For each of these two groups the linear relationship between development time and day of oviposition is indicated as modelled in the random slope lme model (see text). Full random slope lme model fitted was: √(larval mass at 21 days of development) = egg size + day of oviposition + flight treatment + flight treatment*day of oviposition.

Larvae hatching from large eggs were heavier after 21 days of development than larvae that hatched from small eggs (t_193 _= 4.52, p << 0.001). There were no size differences between the eggs laid by control and forced flight females (see earlier), but larvae from control mothers were heavier after 21 days of development than larvae from forced flight mothers (control (± SE) = 48.4 ± 2.2 mg, forced flight (± SE) = 39.2 ± 2.1 mg; flight treatment). There was a significant day of oviposition by flight treatment interaction (t_193 _= 2.64 p = 0.0082; Figure 5). As mothers age, offspring larval mass declines, and this maternal age-specific decline in offspring mass was more pronounced in control compared to forced flight mothers (Figure 5).

#### Survival from the egg stage to 21 days of larval development

Larger eggs had a higher chance of survival (effect coefficient = 4.93, z = 2.83, p = 0.0046).

### Effects of sub-lethal viral infection

#### Survival till pupal stage

On average, the size of larvae allocated to either treatment group (i.e. control vs. viral infection) did not differ (F_1,229 _= 1.56, p = 0.21). Larval mass at day 21 (effect coefficient = 0.022, z = 2.068, p = 0.039), and the viral treatment (effect coefficient = -1.67, z = -3.49, p = 0.00049) had a significant impact on larval survival from day 21 till pupation. Larger larvae had a higher probability of survival, and so did the controls (survival probability, controls (± SE) = 0.94 ± 0.021, infected (± SE) = 0.78 ± 0.042). There were no significant interaction effects.

#### Survival till the adult stage

Additional mortality occurred in the pupal stage, and the only factor that adequately explained this mortality was viral treatment (effect coefficient = -1.45, z = -3.86, p = 0.00011). Control pupae had a higher chance of surviving than the viral treatment pupae (survival probability, controls (± SE) = 0.91 ± 0.025, infected (± SE) = 0.69 ± 0.046).

#### Larval development time

**Figure 6:**
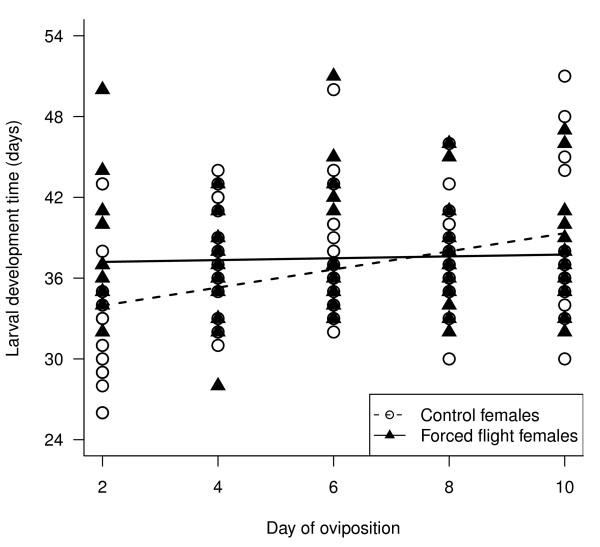
**Total larval development time (days) of the larvae that successfully pupated for each of the sampled oviposition days**. Larvae had mothers that either had been forced to fly or were controls as indicated. For each of these two groups the linear relationship between development time and day of oviposition is indicated as modelled in the random slope lme model (see text). Full fitted random slope lme model: √(larval development time) = egg size + day of oviposition + flight treatment + flight treatment*day of oviposition + viral treatment + sex.

Larval development time was significantly influenced by egg size (t_144 _= -2.61, p = 0.010) and an interaction between day of oviposition and flight treatment (t_144 _= -2.69, p = 0.0080; Figure 6). Offspring hatching from large eggs developed faster, and overall those from mothers that had been forced to fly took longer to develop (larval development time in days: control (± SE) = 36.39 ± 0.41, forced flight (± SE) = 37.45 ± 0.42). The significant interaction between day of oviposition and flight treatment is primarily the result of very fast developers from eggs laid by control females early in the oviposition period (Figure 6). Viral treatment had no effect on larval development time (t_144 _= 0.099, p = 0.92). Female offspring developed more slowly than male offspring (t_144 _= -6.49, p << 0.0001; females (± SE) = 38.72 ± 0.44 days; males (± SE) = 35.17 ± 0.40 days).

#### Pupal mass

There was no effect of egg size (t_145 _= 1.26, p = 0.21), day of oviposition (t_145 _= 0.11, p = 0.91), flight treatment (t_145 _= -0.65, p = 0.52) or viral treatment (t_145 _= 0.79, p = 0.43). There was a significant difference between the sexes (t_145 _= -11.77, p < 0.0001); females were heavier than males (females (± SE) = 121.34 ± 1.03 mg; males (± SE) = 104.67 ± 0.96 mg).

## Discussion

Maternal condition can generate resource-related maternal effects through changes in egg provisioning which can greatly influence offspring growth and survival (for reviews see; [1-4]). In the present study, we observed age-related effects on embryonic development time; with eggs laid by old mothers have longer embryonic development times. Maternal flight treatment influenced maternal reproductive output and this study is the first to demonstrate that increased flight during the oviposition period can reduce both maternal fecundity and the quality or composition of eggs. Changes in maternal egg provisioning due to flight treatment had a direct influence on larval mass and development time. Offspring with lower larval masses had reduced survival after exposure to the viral pathogen.

In *P. aegeria*, a decline in reproductive output with maternal age has been previously reported and is hypothesised to result from a decline in maternal resources over time [7,51,52]. In line with these previous studies, we observed that daily fecundity (after an initial peak on day 3 of oviposition) and egg size both decline over time with maternal age. Contrary to previous studies on other insect species, we found that offspring from older mothers did not differ in; survival during the egg and larval stages [5-8], larval development time [9-12] or pupal mass [9,10]. We did find, however, that maternal age significantly influenced embryonic development time; with eggs laid by older mothers having longer embryonic development times. This suggests that age-related changes in maternal condition generate resource-related maternal effects that influence *P. aegeria *embryonic development [1,30].

Many previous studies, across a wide range of taxa, have observed that egg size can influence offspring fitness [1-4]. In the present study, we found that egg size significantly influenced *P. aegeria *offspring development and survival. Offspring from large eggs had significantly shorter embryonic development times, increased larval masses after 21 days of development, shorter larval development times, and higher egg to adult survival. Although larvae from small eggs had longer larval development times, offspring from large and small eggs did not differ in pupal mass. This suggests that offspring from small eggs compensate for a smaller size early in development by growing and feeding for longer (and see [7]).

Forced flight treatment significantly affected female reproductive output. Relative to control females, females forced to fly laid significantly fewer eggs per day, particularly during the first six days of the oviposition period. There was, however, no effect of flight treatment on egg size. This is an interesting finding because a previous study demonstrated that increased flight prior to the onset of oviposition reduces egg size (but not fecundity) in *P. aegeria *[7]. In the present study, however, a much more intensive flight treatment was used and females were stimulated to fly several times across the oviposition period (rather than once prior to the onset of oviposition as in the previous study). A resource allocation trade-off between fecundity and dispersal has been previously demonstrated in range expanding populations of *P. aegeria *[20]. It is possible, therefore, that the intensive flight treatment used during this experiment also resulted in a decreased resource investment to egg production rather than to egg size. Further experiments are required, however, to determine whether these differences observed across studies are due to differences in flight duration (or intensity) *per se*, or due to other context-dependent factors including (amongst others), population-specific differences in response to flight, or across-study differences in the temperature used during the flight treatment and the oviposition period.

Larvae from eggs laid by females forced to fly had significantly smaller masses (at 21 days of development) and longer larval development times. Given that there were no differences in egg size across flight treatment groups, these results strongly indicate egg provisioning differences between flight treatment groups that are over and above those related to egg size *per se*. As far as we are aware, this is the first study to show that flight during the oviposition period can affect reproductive output both in terms of reducing fecundity and in reducing the quality or composition of resources in eggs. This result also indicates the potential for flight-induced changes in maternal egg provisioning to continue exerting effects later in offspring development. There was, however, no effect of maternal flight treatment on pupal mass. This suggests that offspring from forced flight mothers compensate for a smaller size early in development by growing and feeding for longer.

Viral infection negatively affected survival to both pupation and the adult stage. Effective resistance to pathogens is often dependent on resource availability [37], because resource availability improves body condition and also immune system deployment [38]. It is well recorded that lepidopteran larvae can demonstrate developmental resistance to baculovirus infection and this is often attributed to increasing body weights during larval development [53]. In the present study, offspring with a small larval body mass (at 21 days of development and hence at the time of viral inoculation) had a lower survival to pupation, suggesting that these offspring had reduced resistance to AcMNPV. Further experiments are required, however, to distinguish whether this effect on body masses arises through developmental resistance or through resource allocation to the immune system. Although flight-induced changes in maternal egg provisioning did result in larvae with small masses (at 21 days of development and hence at the time of viral inoculation) we found no significant main effect of flight linking the impact of maternal flight on larval mass. An absence of a direct effect of flight *per se *may not mean, however, that this maternal effect has no importance from an ecological perspective because the net effect is still a reduction in offspring fitness. As such, given the higher susceptibility of smaller larvae to viral infection it would be anticipated that there would be an indirect effect of maternal flight on offspring survival.

Viral infection had no detectable sub-lethal effects on larval development time and pupal mass. There are inconsistencies in studies of the sub-lethal effects of baculoviruses on lepidopteran larval development rate with some demonstrating little or no effect when early instars are treated (e.g.[54,55]) whilst others demonstrate significant effects [32-36]. Contrary to our expectations, relative to control larvae, infected larvae did not grow for longer to gain sufficient resources for both immune system deployment and to obtain the critical mass required for pupation. This result probably reflects a reduction in variation in offspring phenotypes due to differential survival at each stage of development; larvae from small/poorly provisioned eggs fail to hatch, small larvae die during early larval development, more small larvae then die due to viral infection, leaving only the most robust, faster growing larvae to complete their development. Overall, this leads to the conclusion that although a decrease in maternal egg provisioning does reduce larval mass, which in turn decreases offspring survival probability, this reduction in mass does not necessarily confer an *additional *sub-lethal cost to larvae when they are exposed to a viral infection.

## Conclusion

This study clearly demonstrates that in *P. aegeria*, changes in maternal condition due to age, or increased flight during the oviposition period, can generate resource-related maternal effects with consequences for offspring performance across the whole of development from egg to adulthood. In some species, activation of the immune system can influence adult dispersal, particularly if immune system activation indicates a high infection risk in the present habitat [56]. *Pararge aegeria *has attracted attention in studies of distribution and dispersal in response to climate change (e.g. [57]), and in studies of evolutionary trade-offs at both expanding range margins (e.g. [58,59]) and in relation to habitat fragmentation (e.g. [60]). Although duration of our forced flight is longer than estimates from previous studies of average female *P. aegeria *flight periods in the field (12-90 s; for a detailed discussion of flight in female *P. aegeria *see [24]), it remains difficult to determine exactly how relevant our flight treatment is for drawing conclusions about the cost of flight in nature. What is clear from our results, however, is that even a small increase in flight during the oviposition period induces variation in resource allocation to reproduction which generates cross-generational maternal effects that have the potential to influence population dynamics [61]. We suggest, therefore, that more consideration needs to be given to the role that flight-induced maternal effects may play in these ecological processes [61].

## Authors' contributions

MG conceived of the study, carried out the experiments and drafted the manuscript. CJB performed the statistical analyses and helped draft the manuscript. HH designed the inoculation method, carried out the baculovirus production and helped draft the manuscript. RSH provided statistical advice and helped draft the manuscript. HVD coordinated and helped draft the manuscript. All authors participated in the design of the study and read and approved the final manuscript.
